# Effects of Atherogenic Factors on Endothelial Cells: Bioinformatics Analysis of Differentially Expressed Genes and Signaling Pathways

**DOI:** 10.3390/biomedicines11041216

**Published:** 2023-04-19

**Authors:** Stanislav Kotlyarov

**Affiliations:** Department of Nursing, Ryazan State Medical University, 390026 Ryazan, Russia; skmr1@yandex.ru

**Keywords:** atherosclerosis, endothelial cells, gene ontology, risk factors, smoking, hemodynamics, oxidized low-density lipoprotein

## Abstract

(1) Background: Atherosclerosis is a serious medical condition associated with high morbidity and mortality rates. It develops over many years as a complex chain of events in the vascular wall involving various cells and is influenced by many factors of clinical interest. (2) Methods: In this study, we performed a bioinformatic analysis of Gene Expression Omnibus (GEO) datasets to investigate the gene ontology of differentially expressed genes (DEGs) in endothelial cells exposed to atherogenic factors such as tobacco smoking, oscillatory shear, and oxidized low-density lipoproteins (oxLDL). DEGs were identified using the limma R package, and gene ontology (GO), Kyoto Encyclopedia of Genes and Genomes (KEGG) pathway enrichment, and protein–protein interaction (PPI) network analysis were performed. (3) Results: We studied biological processes and signaling pathways involving DEGs in endothelial cells under the influence of atherogenic factors. GO enrichment analysis demonstrated that the DEGs were mainly involved in cytokine-mediated signaling pathway, innate immune response, lipid biosynthetic process, 5-lipoxygenase activity, and nitric-oxide synthase activity. KEGG pathway enrichment analysis showed that common pathways included tumor necrosis factor signaling pathway, NF-κB signaling pathway, NOD-like receptor signaling pathway, lipid and atherosclerosis, lipoprotein particle binding, and apoptosis. (4) Conclusions: Atherogenic factors such as smoking, impaired flow, and oxLDL contribute to impaired innate immune response, metabolism, and apoptosis in endothelial cells, potentially leading to the development of atherosclerosis.

## 1. Introduction

Atherosclerosis is a major problem in modern society because of the high prevalence of atherosclerotic cardiovascular diseases (ASCVDs) among populations in many countries [1]. ASCVDs are an important cause of health care seeking, hospitalization, and mortality [2]. It is important to note that ASCVDs are often diagnosed belatedly, when cardiovascular complications are already present and when the effectiveness of therapeutic interventions may be insufficient. These and other data reinforce the significance of the prevention and early diagnosis of atherosclerosis.

Atherosclerosis is an actively studied problem that has allowed the accumulation of extensive data concerning the different stages of atherogenesis [3]. Atherosclerosis develops in the vascular wall over several years and is the result of a complex chain of events, many of which are cross-linked. These processes involve many cells in both the vascular wall and the peripheral blood flow. Endothelial cells are key participants in atherogenesis and have been the subject of numerous studies [4]. A growing body of data reinforces the understanding that the structure and function of endothelial cells are much more complex than simply providing a barrier between blood flow and the vascular wall. Endothelial cells cover the entire vascular bed in a monolayer, regulate the permeability of substances and cells in the tissue, and perform many other functions, such as regulating blood flow, regulating the behavior of other cells in the vascular wall and blood flow, immune function, and participation in hemostasis. In addition, recent data allow endothelial cells to be considered as coordinating cells that perform many of these functions.

Endothelial dysfunction, which can result from various factors affecting the endothelium, is a critical link in the pathogenesis of atherosclerosis [5,6]. Dyslipidemia and smoking are known risk factors for endothelial dysfunction [7,8]. In addition to these systemic factors, local hemodynamic disturbances are important atherogenic factors [5,9]. Disturbances in laminar blood flow observed at arterial bifurcations or branches contribute to the development of atherosclerosis [10,11].

Bioinformatics analysis methods have become increasingly important in recent years due to the vast amount of genomic data that is being generated. One of the most valuable resources for genomic data is the Gene Expression Omnibus (GEO) [12], a public repository of high-throughput sequencing data hosted by the National Center for Biotechnology Information (NCBI). Bioinformatics analysis methods play a crucial role in making sense of the vast amount of data stored in the GEO. These methods are used to identify differentially expressed genes (DEGs), perform gene ontology (GO) analysis, and perform pathway analysis. In addition, bioinformatics methods can be used to integrate data from different sources to gain a more comprehensive understanding of the biological processes underlying a particular disease or condition.

Thus, the aim of the present study was to investigate the biological processes and signaling pathways associated with the effects of factors such as oxidized low-density lipoprotein (oxLDL), tobacco smoke extract, and hemodynamic disturbances (oscillatory shear) on endothelial cells using bioinformatics analysis.

## 2. Materials and Methods

### 2.1. Data Collection

Publicly available sets containing information on gene expression in endothelial cells exposed to the following factors under laboratory conditions were used as the data source for the analysis: tobacco smoke extract, oxLDL, and oscillatory flow versus pulsatile flow. The analysis was performed on datasets obtained from the GEO, NCBI. The Gene Expression Omnibus (GEO) is a web-based database containing gene expression data, hybridization arrays, chips, and microarrays. Datasets for analysis were searched using the keywords “endothelial cells”, “tobacco smoke”, “oscillatory shear”, and “oxidized LDL”. The search was limited to human studies and studies that used gene expression profiling data. According to the search criteria, the following datasets were selected for analysis: GSE141136, GSE103672, and GSE29881.

For the GSE141136 dataset, we analyzed gene expression levels in human endothelial cells exposed to tobacco smoke extract [13]. Data were obtained using a GPL11154 Illumina HiSeq 2000 platform (Homo sapiens). The comparison groups were human aorta endothelial cells (HAECs) and human coronary artery endothelial cells (HCAECs) exposed to 6 h of tobacco smoke extract and cells similar to the control group.

For the GSE103672 dataset, we extracted samples obtained by exposing human umbilical vein endothelial cells (HUVECs) to oscillatory shear (OS) (0.5 ± 5 dyn/cm^2^) versus pulsatile shear (PS) (12 ± 5 dyn/cm^2^) [14]. Data were obtained using a GPL11154 Illumina HiSeq 2000 platform.

For the GSE29881 dataset, we analyzed gene expression levels in human umbilical artery endothelial cells (HUAECs) in response to oxLDL [15]. Data were obtained using the GPL570 [HG-U133_Plus_2] Affymetrix Human Genome U133 Plus 2.0 Array platform.

### 2.2. Differential Expression Analysis

The limma package in Bioconductor R (v. 4.0.2) [16] was used to identify differentially expressed genes in the comparison groups, using log2 transformation and quantile normalization for data normalization. The level of statistical significance for multiple comparisons was adjusted using the Benjamini and Hochberg (false discovery rate, FDR) algorithm. For the GSE141136 and GSE103672 datasets, the screening conditions for differentially expressed genes were an absolute value of logFC > 1.5 and *p* values satisfying the FDR condition ≤ 5%, while for the GSE29881 dataset, the screening conditions were logFC > 1.5 and *p* values satisfying the condition *p* ≤ 0.05.

Protein–protein interactions (PPIs) of protein products of DEGs were assessed using the online tool Search Tool for the Retrieval of Interacting Genes database (STRING) [17]. Relationships between DEGs were analyzed using the Network Analyzer plug-in module of Cytoscape software [18,19]. Molecular Complex Detection (MCODE v. 2.0.2) was used to identify the gene clusters in the PPI network [20].

Furthermore, the cytoHubba application in Cytoscape software [21] was used to identify the most important genes (hub genes) in the network by ranking the nodes in the network according to their network characteristics. The maximal clique centrality (MCC) topological analysis algorithm was used to analyze, predict, and visualize key proteins in molecular PPI interaction networks.

Next, the analysis of differential expression of chemokine genes in the datasets of the comparison groups was performed. The conditions for screening differentially expressed genes for the GSE141136 and GSE103672 datasets were *p* values that met the FDR condition ≤ 5%, and for the GSE29881 dataset, *p* values that met the *p* ≤ 0.05 condition. Visualization of gene expression levels in the comparison groups in each dataset was performed using the Phantasus application (v. 1.19.3) [22]. Data were visualized as box plots.

### 2.3. Gene Ontology Analysis

Gene ontology analysis was performed to identify biological processes and Kyoto Encyclopedia of Genes and Genomes (KEGG) pathways for the DEGs in the network [23,24,25]. This analysis was performed using GEO2Enrichr [26], ShinyGO v0.77 [27], and g:Profiler [28], which derive biological processes from the Gene Ontology (GO) Resource gene consortium database [29,30,31,32]. The signaling pathways were identified using the KEGG and Reactome databases [33], and their functional enrichment and visualization were established using a *p* < 0.05 value corrected using the Benjamini and Hochberg algorithm as the threshold. The data were visualized using an online platform for data analysis and visualization: http://www.bioinformatics.com.cn/srplot (accessed on 1 March 2023).

## 3. Results

### 3.1. Identification and Functional Annotation of DEGs in Endothelial Cells during Flow Changes

**Figure 1:**
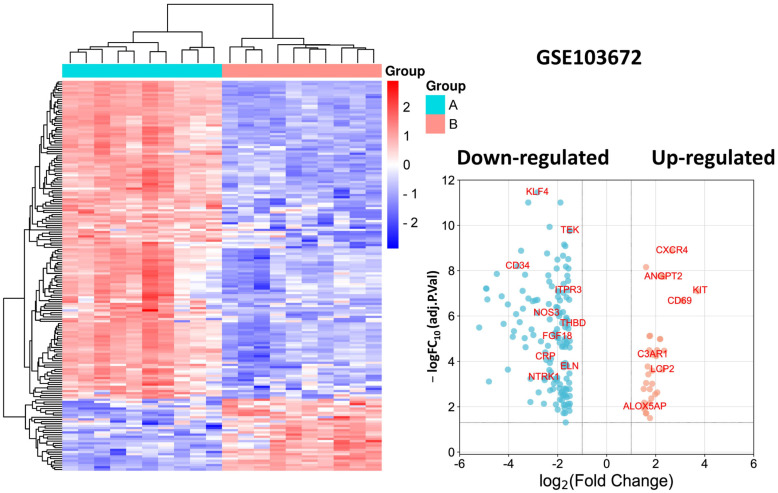
Heatmap and volcano plot for DEGs from dataset GSE103672. Note: the identified hub DEGs are shown in the volcano plot.

In the GSE103672 dataset, we identified a total of 179 genes that were differentially expressed between endothelial cells (HAECs and HCAECs) exposed to OS compared with PS for 9 to 24 h, with 33 genes up-regulated and 146 genes down-regulated (Figure 1, Supplementary Figures S1 and S2).

The heatmap shows the expression levels of all DEGs, with red indicating up-regulation and blue indicating down-regulation. The volcano plot shows the fold change (log2) of each gene plotted against its statistical significance (−log10 FDR *p* value) in a scatter plot, with significant genes colored in red. The heatmap and volcano plot provide a visualization of the gene expression patterns and identify genes that are differentially expressed under OS conditions.

**Figure 2:**
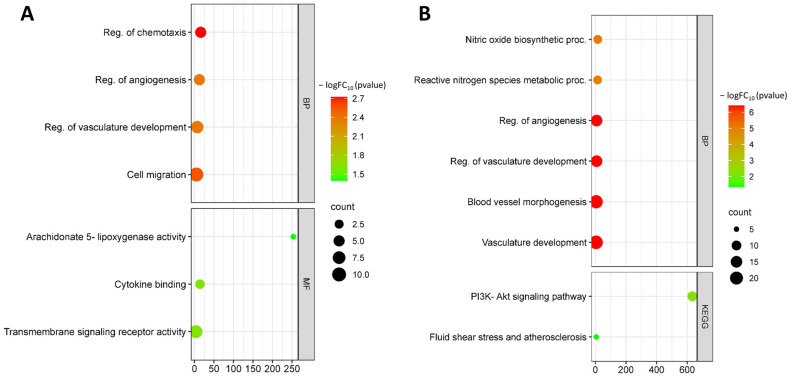
Functional enrichment of up-regulated DEGs (**A**) and down-regulated DEGs (**B**) from the GSE103672 dataset.

Functional enrichment analysis by biological processes showed that up-regulated DEGs under OS are involved in biological processes such as the regulation of chemotaxis (GO:0050920), chemotaxis (GO:0006935), regulation of angiogenesis (GO:0045765), vasculature development (GO:0001944), and cell migration (GO:0016477). Up-regulated DEGs are involved in molecular functions such as arachidonate 5-lipoxygenase activity (GO:0004051), cytokine binding (GO:0019955), immune receptor activity (GO:0140375), and transmembrane signaling receptor activity (GO:0004888) (Figure 2A).

Down-regulated DEGs are involved in such processes as nitric oxide biosynthetic process (GO:0006809), reactive nitrogen species metabolic process (GO:2001057), positive regulation of angiogenesis (GO:0045766), blood vessel morphogenesis (GO:0048514), blood circulation (GO:0008015), and regulation of cell population proliferation (GO:0042127). Down-regulated DEGs are involved in KEGG pathways such as fluid shear stress and atherosclerosis (hsa05418) and Phosphatidylinositol 3-kinase (PI3K)-Akt signaling pathway (hsa04151) (Figure 2B).

**Figure 3:**
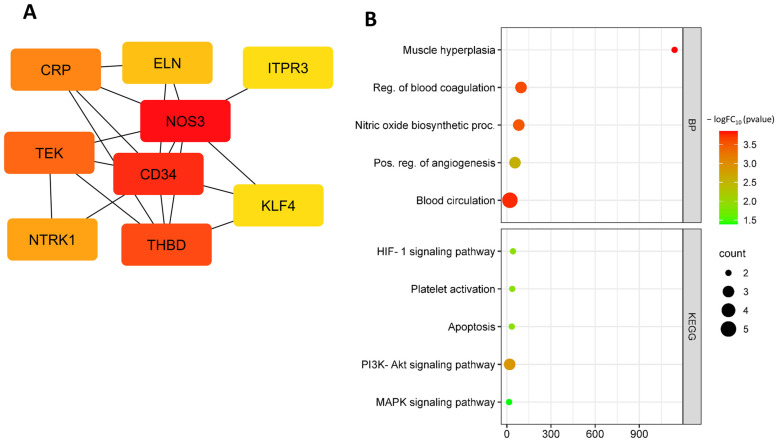
Down-regulated hub DEGs at OS (**A**) and functional enrichment of down-regulated hub DEGs at OS (**B**).

Further analysis identified the following down-regulated hub DEGs: *CRP*, *TEK*, *NTRK1*, *CD34*, *FGF18*, *NOS3*, *KLF4*, *THBD*, *ITPR3*, and *ELN* (Figure 3A, Table 1). Down-regulated hub DEGs are involved in biological processes such as the regulation of muscle hyperplasia (GO:0014738), negative regulation of blood coagulation (GO:0030195), positive regulation of angiogenesis (GO:0045766), blood circulation (GO:0008015), nitric oxide biosynthetic process (GO:0006809), blood coagulation (GO:0007596), and response to fluid shear stress (GO:0034405 (Figure 3A)). Down-regulated hub DEGs are involved in KEGG pathways such as hypoxia-inducible factor-1 (HIF-1) signaling pathway (hsa04066), inflammatory mediator regulation of TRP channels (hsa04750), platelet activation (hsa04611), apoptosis (hsa04210), fluid shear stress and atherosclerosis (hsa05418), ATP-binding cassette (ABC) transporters (hsa02010), PI3K-Akt signaling pathway (hsa04151), and mitogen-activated protein kinase (MAPK) signaling pathway (hsa04010) (Figure 3B).

**Figure 4:**
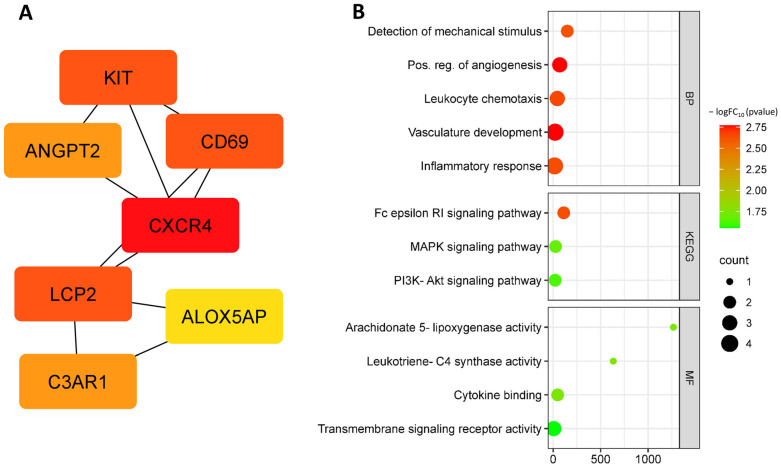
Up-regulated hub DEGs at OS (**A**) and functional enrichment by biological processes of up-regulated hub DEGs at OS (**B**).

Further analysis identified the following up-regulated hub DEGs: *ALOX5AP*, *ANGPT2*, *KIT*, *LCP2*, *C3AR1*, *CXCR4*, and *CD69* (Figure 4A, Table 2). These DEGs are involved in such biological processes as the detection of mechanical stimulus involved in sensory perception (GO:0050974), detection of mechanical stimulus (GO:0050982), leukocyte chemotaxis (GO:0030595), inflammatory response (GO:0006954), blood vessel morphogenesis (GO:0048514), myeloid leukocyte activation (GO:0002274), and leukotriene production involved in inflammatory response (GO:0002540) (Figure 4B).

Thus, oscillating shear (OS) may contribute to changes in gene expression in endothelial cells associated with the development of atherosclerosis.

### 3.2. Identification and Functional Annotation of DEGs in Endothelial Cells in Smoking

**Figure 5:**
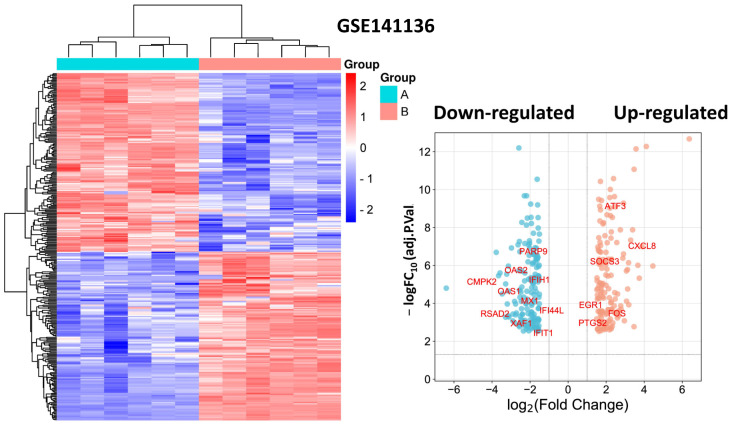
Heatmap and volcano plot for DEGs from dataset GSE141136. Note: the identified hub DEGs are shown in the volcano plot.

In the GSE141136 dataset, we identified a total of 303 genes that were differentially expressed between endothelial cells (HAECs and HCAECs) exposed to tobacco smoke extract and controls, with 147 genes up-regulated and 156 genes down-regulated (Figure 5, Supplementary Figures S3 and S4). We performed functional annotation of the identified differentially expressed genes using GO and KEGG pathway analysis.

**Figure 6:**
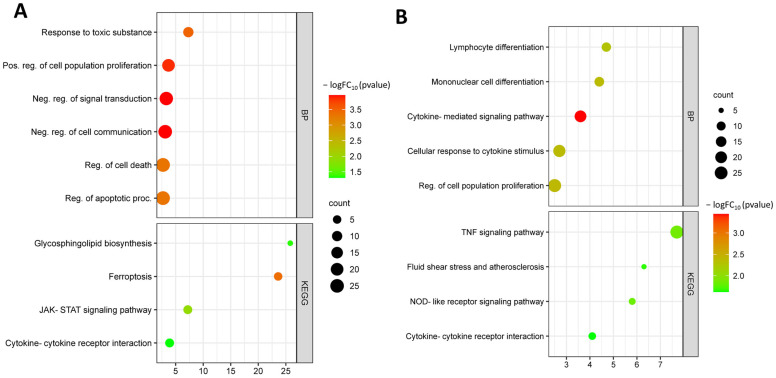
Functional enrichment of up-regulated DEGs from dataset GSE141136 (**A**); functional enrichment of down-regulated DEGs from dataset GSE141136 (**B**).

Functional enrichment analysis by biological processes showed that up-regulated DEGs were involved in biological processes such as response to toxic substance (GO:0009636), positive regulation of cell population proliferation (GO:0008284), negative regulation of signal transduction (GO:0009968), regulation of cell death (GO:0010941), and regulation of apoptotic process (GO:0042981). Up-regulated DEGs are involved in KEGG pathways such as glycosphingolipid biosynthesis (hsa00604), ferroptosis (hsa04216), cytokine–cytokine receptor interaction (hsa04060) (Figure 6A).

Down-regulated DEGs from this dataset were involved in biological processes such as lymphocyte differentiation (GO:0030098), mononuclear cell differentiation (GO:1903131), cytokine-mediated signaling pathway (GO:0019221), cellular response to cytokine stimulus (GO:0071345), and regulation of cell population proliferation (GO:0042127). Down-regulated DEGs were involved in the KEGG pathway: TNF signaling pathway (hsa04668), fluid shear stress and atherosclerosis (hsa05418), NOD-like receptor signaling pathway (hsa04621), and cytokine–cytokine receptor interaction (hsa04060) (Figure 6B).

**Figure 7:**
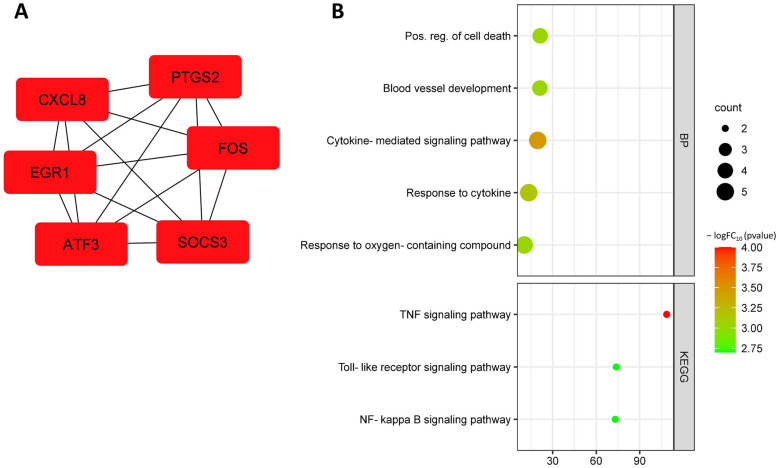
Up-regulated hub DEGs from dataset GSE141136 (**A**); functional enrichment of up-regulated hub DEGs from dataset GSE141136 (**B**).

Further analysis identified the following up-regulated hub DEGs: *EGR1*, *ATF3*, *CXCL8*, *SOCS3*, *FOS*, and *PTGS2* (Figure 7A, Table 3). Up-regulated hub DEGs are involved in the following biological processes: positive regulation of cell death (GO:0010942), blood vessel development (GO:0001568), cytokine-mediated signaling pathway (GO:0019221), cellular response to oxygen-containing compounds (GO:1901701), response to cytokine (GO:0034097), and negative regulation of cell communication (GO:0010648). Up-regulated hub DEGs are involved in KEGG pathways such as TNF signaling pathway (hsa04668), toll-like receptor signaling pathway (hsa04620), and NF-kappa B signaling pathway (hsa04064) (Figure 7B).

**Figure 8:**
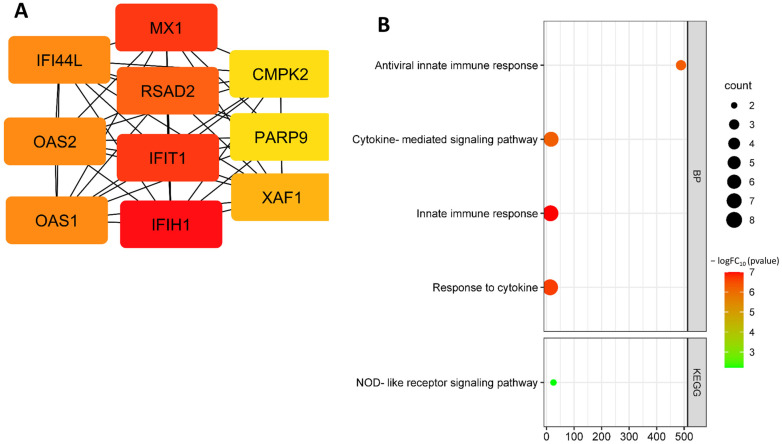
Down-regulated hub DEGs from dataset GSE141136 (**A**); functional enrichment of down-regulated hub DEGs from dataset GSE141136 (**B**). Note: the most important genes are ranked as follows: the most important genes are highlighted in red, less important genes in orange, even less important genes in yellow.

The down-regulated hub DEG search identified the following genes: *MX1*, *CMPK2*, *IFIT1*, *OAS2*, *IFI44L*, *IFIH1*, *PARP9*, *RSAD2*, *XAF1*, and *OAS1* (Figure 8A, Table 4). Down-regulated hub DEGs are involved in the following biological processes: antiviral innate immune response (GO:0140374), cytokine-mediated signaling pathway (GO:0019221), innate immune response (GO:0045087), and response to cytokine (GO:0034097) (Figure 8B).

Thus, smoking may contribute to changes in gene expression in endothelial cells associated with the development of atherosclerosis, including regulation of the innate immune response, such as the TNF signaling pathway, toll-like receptor signaling pathway, NF-kappa B signaling pathway, and cytokine-mediated signaling pathway, regulation of cell death, and cellular response to oxygen-containing compounds.

### 3.3. Identification and Functional Annotation of DEGs in Endothelial Cells upon Exposure to Oxidized LDL

**Figure 9:**
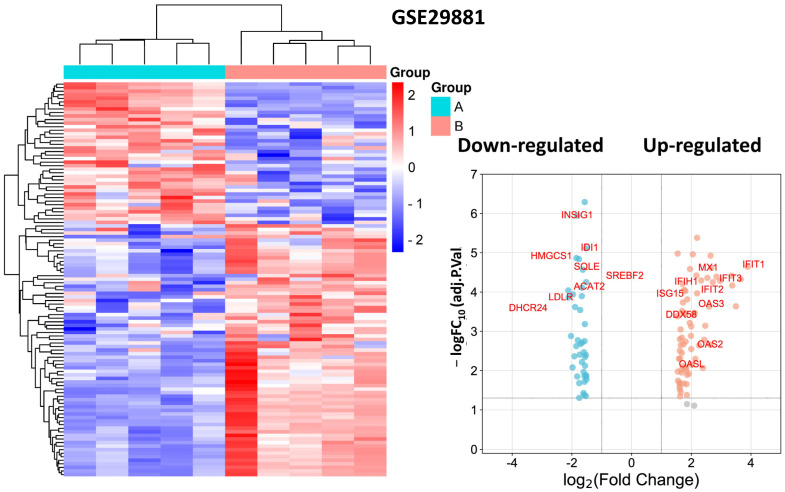
Heatmap and volcano plot for DEGs from dataset GSE29881. Note: the identified hub DEGs are shown in the volcano plot.

We identified a total of 112 genes that were differentially expressed between oxLDL treatment and control, with 71 up-regulated and 41 down-regulated genes from the GSE29881 dataset (Figure 9, Supplementary Figures S5 and S6).

**Figure 10:**
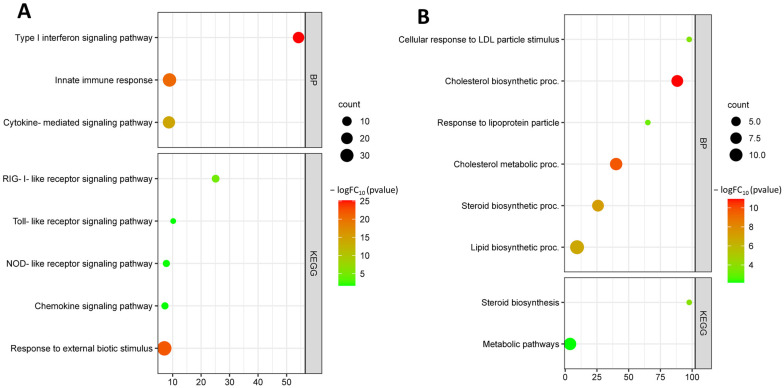
Functional enrichment of up-regulated DEGs of the GSE29881 dataset (**A**); functional enrichment of down-regulated DEGs of the GSE29881 dataset (**B**).

The GO analysis revealed that the up-regulated genes were mainly involved in Type I interferon signaling pathway (GO:0060337), innate immune response (GO:0045087), cytokine-mediated signaling pathway (GO:0019221), response to external biotic stimulus (GO:0043207), and response to cytokine (GO:0034097). Up-regulated DEGs are involved in KEGG pathways such as RIG-I-like receptor signaling pathway (hsa04622), toll-like receptor signaling pathway (has04620), NOD-like receptor signaling pathwahashsa04621), and chemokine signaling pathway (hsa04062) (Figure 10A).

Down-regulated DEGs are involved in biological processes such as cellular response to low-density lipoprotein particle stimulus (GO:0071404), cholesterol biosynthetic process (GO:0006695), response to lipoprotein particle (GO:0055094), cholesterol metabolic process (GO:0008203), steroid biosynthetic process (GO:0006694), and lipid biosynthetic process (GO:0008610). Down-regulated DEGs are involved in KEGG pathways such as steroid biosynthesis (hsa00100) and metabolic pathways (hsa01100) (Figure 10B).

**Figure 11:**
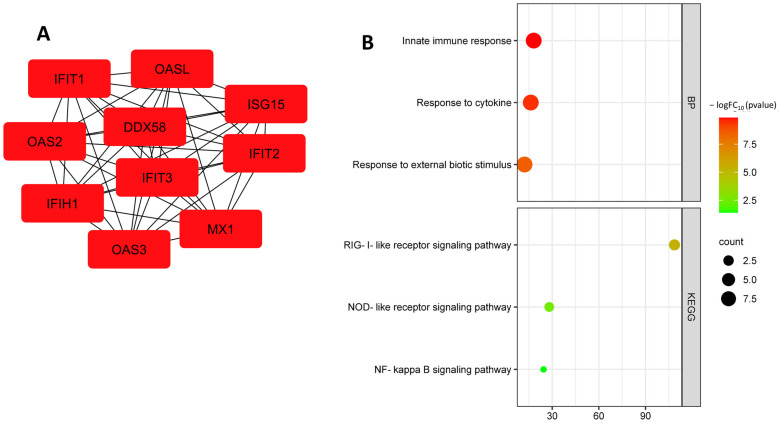
Up-regulated hub DEGs of the GSE29881 dataset (**A**); functional enrichment of the up-regulated hub DEGs of the GSE29881 dataset (**B**).

A search for up-regulated hub DEGs identified the following genes: *IFIH1*, *IFIT1*, *OAS2*, *OAS3*, *DDX58*, *ISG15*, *MX1*, *OASL*, *IFIT3* and *IFIT2* (Figure 11A, Table 5). Up-regulated hub DEGs are involved in biological processes such as Type I interferon signaling pathway (GO:0060337), innate immune response (GO:0045087), response to cytokine (GO:0034097), and response to external biotic stimulus (GO:0043207). Up-regulated hub DEGs are involved in KEGG pathways such as RIG-I-like receptor signaling pathway (hsa04622), NOD-like receptor signaling pathway (hsa04621), and NF-kappa B signaling pathway (hsa04064) (Figure 11B).

**Figure 12:**
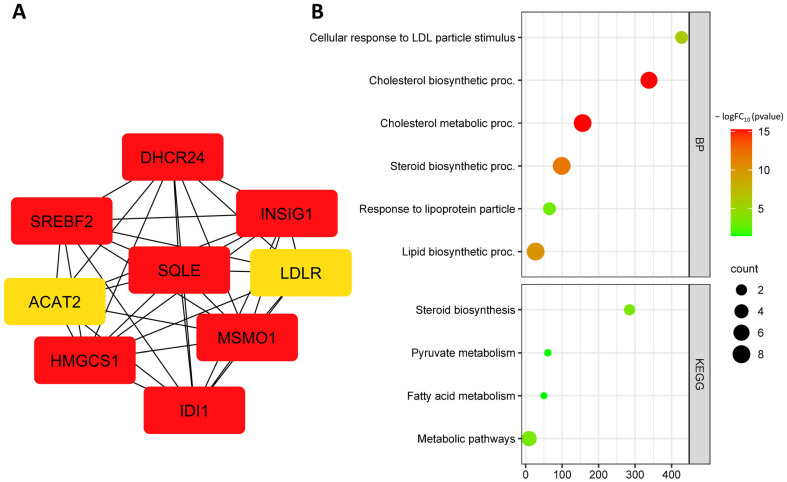
Down-regulated hub DEGs of the GSE29881 dataset (**A**); functional enrichment of down-regulated hub DEGs of the GSE29881 dataset (**B**). Note: the most important genes are ranked as follows: the most important genes are highlighted in red, less important genes in orange, even less important genes in yellow.

A search for down-regulated hub DEGs identified the following genes: *IDI1*, *INSIG1*, *LDLR*, *SREBF2*, *DHCR24*, *HMGCS1*, *ACAT2*, and *SQLE* (Figure 12A, Table 6). Down-regulated hub DEGs are involved in biological processes such as cellular response to low-density lipoprotein particle stimulus (GO:0071404), cholesterol biosynthetic process (GO:0006695), cholesterol metabolic process (GO:0008203), steroid biosynthetic process (GO:0006694), response to lipoprotein particle (GO:0055094), and lipid biosynthetic process (GO:0008610) (Figure 12B). Down-regulated hub DEGs are involved in KEGG pathways such as steroid biosynthesis (hsa00100), pyruvate metabolism (hsa00620), fatty acid metabolism (hsa01212), peroxisome proliferator-activated receptor (PPAR) signaling pathway (hsa03320), and metabolic pathways (hsa01100) (Figure 12B).

### 3.4. Identification and Functional Annotation of Common DEGs in Endothelial Cells under Different Risk Factors

**Figure 13:**
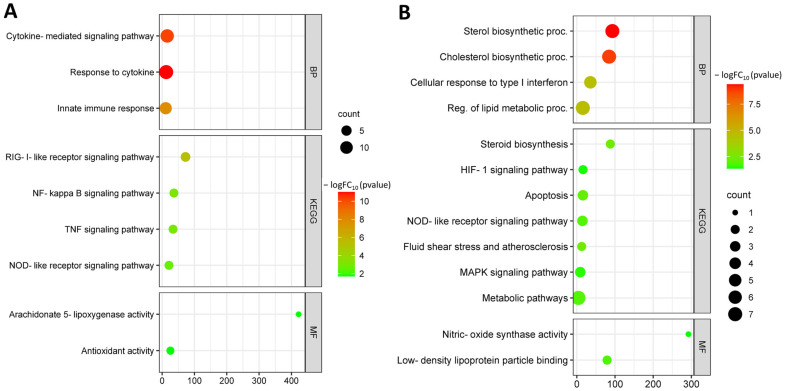
Functional enrichment of common up-regulated hub DEGs (**A**); functional enrichment of common down-regulated hub DEGs (**B**).

Functional enrichment of common hub DEGs from the GSE103672, GSE141136, and GSE29881 datasets were analyzed. Up-regulated common hub DEGs are involved in biological processes such as cytokine-mediated signaling pathway (GO:0019221), response to cytokine (GO:0034097), and innate immune response (GO:0045087). Up-regulated hub DEGs are involved in molecular functions such as arachidonate 5-lipoxygenase activity (GO:0004051), chemokine activity (GO:0008009), leukotriene-C4 synthase activity (GO:0004464), and antioxidant activity (GO:0016209). Up-regulated hub DEGs are involved in KEGG pathways such as RIG-I-like receptor signaling pathway (hsa04622), NF-kappa B signaling pathway (hsa04064), TNF signaling pathway (hsa04668), and NOD-like receptor signaling pathway (hsa04621) (Figure 13A).

Down-regulated common hub DEGs were involved in biological processes such as cholesterol biosynthetic process (GO:0006695), protein targeting to ER (GO:0045047), translational initiation (GO:0006413), and lipid biosynthetic process (GO:0008610). Down-regulated hub DEGs are involved in molecular functions such as nitric-oxide synthase activity (GO:0004517), low-density lipoprotein particle binding (GO:0030169), and lipoprotein particle binding (GO:0071813). Down-regulated hub DEGs are involved in KEGG pathways such as steroid biosynthesis (hsa00100), HIF-1 signaling pathway (hsa04066), NOD-like receptor signaling pathway (hsa04621), apoptosis (hsa04210), fluid shear stress and atherosclerosis (hsa05418), lipid and atherosclerosis (hsa05417), and MAPK signaling pathway (hsa04010) (Figure 13B).

Thus, the most significant DEGs obtained from the three sets are involved in the innate immune system, metabolic regulation, and hemodynamics.

### 3.5. Identification of Differentially Expressed Genes of Chemokines

**Figure 14:**
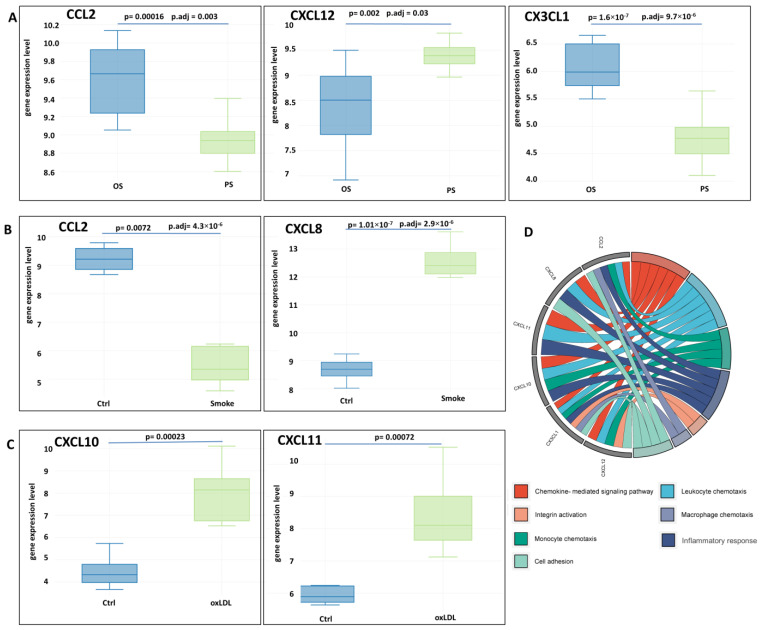
Patterns of chemokine gene expression in endothelial cells in the comparison groups ((**A**) GSE103672; (**B**) GSE141136; (**C**) GSE29881) associated with exposure to risk factors. (**D**) Gene ontology analysis by biological process for differentially expressed chemokine genes in datasets. Note: the data are visualized as box plots (**A**–**C**). Statistically significant differences (*p* values and *p* values corrected with the Benjamini and Hochberg (adj.P.Val) algorithm) are shown between comparison groups.

Using the cutoff criteria for each of the datasets, the following chemokine DEGs were identified (Figure 14). *CCL2*, *CXCL12*, and *CX3CL1* expression was up-regulated in oscillatory shear compared to pulsatile shear. Endothelial cells exposed to tobacco smoke had up-regulated *CXCL8* gene expression and down-regulated *CCL2. CXCL10* and *CXCL11* gene expression was up-regulated in endothelial cells exposed to oxLDL (Figure 14). The identified differentially expressed chemokine genes are involved in chemotaxis, cell adhesion, integrin activation, and inflammatory response (Figure 14D).

The data obtained suggest a differential contribution of the studied risk factors to the expression of chemokines, which may lead to the disruption of the normal immune balance in the endothelium during atherogenesis.

## 4. Discussion

**Figure 15:**
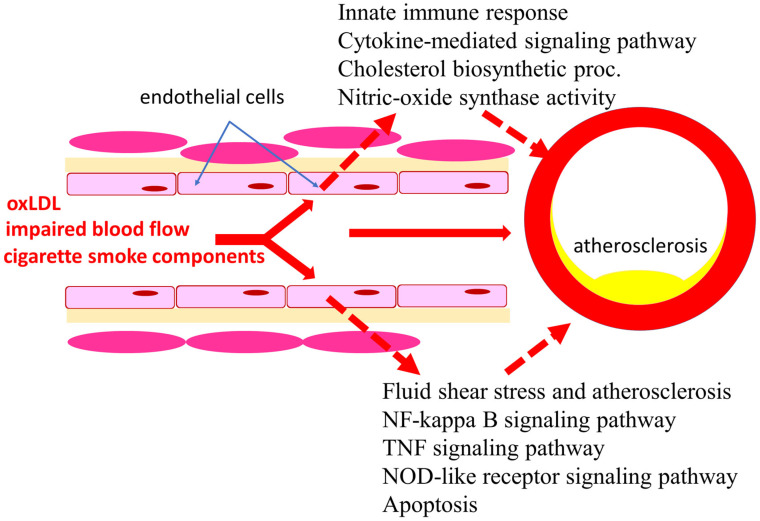
Significance of risk factors in the development of atherosclerosis.

The present study aimed to analyze the functional enrichment of differentially expressed genes in endothelial cells exposed to several key risk factors, including tobacco smoke extract, oxLDL, and OS. Datasets from the GEO, NCBI, were used for the analysis. The results indicated that exposure of endothelial cells to these risk factors could potentially affect the innate immune response, metabolism, and apoptosis in endothelial cells, which could contribute to the development of atherosclerosis (Figure 15).

Atherosclerosis is a chronic inflammatory disease characterized by the accumulation of lipids and immune cells in the arterial wall and the development of atherosclerotic plaques. Evaluating the molecular mechanisms of atherogenic risk factors, such as smoking and dyslipidemia, is critical for understanding the basic pathophysiology of atherosclerosis.

Smoking and dyslipidemia are major risk factors for atherosclerosis [34,35]. Smoking is associated with several atherogenic mechanisms: it increases oxidative stress and causes inflammation, endothelial dysfunction, and immune cell activation. Dyslipidemia is characterized by impaired lipid metabolism, leading to an increase in LDL cholesterol and a decrease in high-density lipoprotein cholesterol (HDL). This leads to the accumulation of LDL cholesterol in the arterial walls, where it is oxidized and taken up by macrophages to form foam cells that are involved in plaque formation [36]. In addition to these systemic risk factors, local hemodynamic disturbances contribute to the development of atherosclerotic lesions in certain arterial areas [37,38].

Evaluation of the molecular mechanisms underlying these risk factors is important to identify potential targets for therapy. Inflammation plays a critical role in the development of atherosclerosis, the underlying cause of cardiovascular disease. Endothelial cells, which line the inner surface of blood vessels, are key players in the initiation and progression of atherosclerosis. In addition to their role in maintaining vascular homeostasis, endothelial cells are involved in the innate immune response through the secretion of cytokines and chemokines, expression of adhesion molecules, and activation of immune cells.

Endothelial dysfunction is an important step in atherogenesis because the endothelium plays a key role in vascular biology, and impaired NO-mediated vasodilation is considered a critical factor in disease development and progression. Endothelial dysfunction develops as a result of a chain of events related to the effects of systemic inflammation and oxidative stress from smoking, dyslipidemia, and is also associated with local hemodynamic disturbances.

Hemodynamic disturbances are considered to be an important factor involved in the early stages of atherogenesis and largely determine the localization of atherosclerotic lesions. Shear stress is considered a key hemodynamic flow characteristic, which is detected by endothelial cells. High shear stress levels are thought to be atheroprotective, whereas low shear stress levels promote atherogenesis. Endothelial cells can detect and respond to these changes through the production of vasoactive substances such as nitric oxide (NO). NO is a potent vasodilator that plays an important role in maintaining endothelial cell function and preventing atherosclerosis [4,39,40]. A key enzyme involved in NO synthesis is endothelial nitric oxide synthase (eNOS). In the current study, an oscillatory shift was shown to contribute to the decreased expression of the *NOS3* gene encoding eNOS. In addition, the current study found that oscillatory shear decreased the expression of the *ASS1* gene. This gene encodes argininosuccinate synthetase 1 (ASS1), an enzyme that regulates the production of l-arginine, which serves as a substrate for eNOS, thereby regulating nitric oxide production and monocyte adhesion [41]. Thus, disruption of laminar flow acting through NOS3 and ASS1 may contribute to disruption of NO-mediated vasodilation and monocyte adhesion.

Another endothelial mechanism of hemodynamic regulation is prostaglandin I2 synthase, encoded by the *PTGIS* gene. In the current study, *PTGIS* expression was found to decrease during oscillatory flow. Prostaglandin I2 synthase biosynthesizes prostaglandin I2 (PGI2), also known as prostacyclin [42]. PGI2 is a potent vasodilator and platelet aggregation inhibitor that plays an important role in the regulation of the circulatory and immune systems. It is known that *PTGIS* expression and PGI2 production are associated with shear stress and that PGI2 deficiency may contribute to the development of atherosclerosis. These data improve the understanding of the role of hemodynamic disturbances in the regulation of endothelial vasoactive substance production and atherogenesis.

It is also known that endothelial cells respond to flow disturbances with certain structural changes that correspond to some metabolic changes. The detection and transduction of flow disturbances involves complex mechanisms. The current study showed that flow disturbances can affect the expression of the Kruppel-like factors KLF4 and KLF2, which are an important link between blood flow disturbances and endothelial cell function [43,44]. KLF4 is a transcription factor involved in various cellular processes, including differentiation, proliferation, and apoptosis. It regulates the expression of genes involved in inflammation, oxidative stress, and lipid metabolism, which are important processes in atherogenesis. KLF4 also plays an important role in endothelial cell differentiation by regulating the expression of genes involved in endothelial cell function and angiogenesis [45,46,47]. Overexpression of Klf4 increases the expression of several anti-inflammatory and antithrombotic factors in endothelial cells. Klf4 also inhibits TNF-induced expression of vascular cell adhesion molecule-1 (Vcam1) by blocking the binding of nuclear factor-κB to the Vcam1 promoter [48]. The expression of these genes can promote the attraction of leukocytes to the arterial wall, leading to the formation of atherosclerotic plaques. On the other hand, KLF4 may be involved in the regulation of proliferation and differentiation of vascular smooth muscle cells (VSMCs), acting as a “molecular switch” of their function [49,50,51].

Another important function of KLF4 is related to its involvement in the regulation of the endothelial cell cytoskeleton. KLF4 regulates the function of several cytoskeletal proteins and also regulates the expression of proteins involved in the formation and maintenance of endothelial cell junctions. KLF4 has been shown to regulate the expression of VE-cadherin, which contributes to the maintenance of the endothelial barrier [52].

Another important function of KLF4 is its involvement in cross-linking with cellular metabolism [53]. Glycolysis is known to be the major energy source for endothelial cells, producing up to 85% of ATP [54,55,56,57]. This occurs despite the fact that endothelial cells have access to oxygen from the blood, and under conditions of migration, proliferation or stress, endothelial cells further increase glycolysis to meet energy demands. Increased glycolysis is a factor in the immune metabolism of endothelial cells, as glycolysis provides cells with energy and metabolites for the immune response. High levels of glycolysis in endothelial cells are regulated by the actions of hexokinase 2 (HK2) and phosphofructokinase 1 (PFK1). In turn, PFK1 activity is controlled by PFKFB3, an enzyme that produces fructose 2,6-bisphosphate (F2,6P2), which serves as an allosteric activator of PFK1 [58,59]. KLF4, in turn, functions as a transcriptional repressor of PFKFB3 and may inhibit inflammation by normalizing glycolysis [60]. Another member of the KLF family, KLF2, whose expression is also increased by laminar flow, represses the activity of the PFKFB3 promoter so that shear-stress-mediated repression of endothelial cell metabolism controls their phenotype [61]. Thus, laminar shear stress, acting through KLF2 and KLF4, reduces the expression of key glycolytic enzymes, such as PFKFB3, resulting in decreased glycolysis and reduced proinflammatory endothelial cell activity. The data obtained in the present study showed a decrease in KLF4 and KLF2 gene expression during oscillatory flow, which may act as a link between vascular hemodynamics and cellular immunometabolism important in atherogenesis.

In addition, other mechanisms of the hemodynamic-inflammatory relationship involving KLF4 are of interest. Endothelial KLF4 regulates Notch expression and activity. Notch signaling pathways play an important role in the transduction of laminar shear stress [62]. NOTCH1 has been shown to sense changes in flow and is essential for the endothelial cell response to flow [63]. Loss of endothelial Notch1 in vivo leads to inflammation [63].

It is also known that impaired hemodynamics can affect endothelial barrier function, which is an important step in atherogenesis. The current study demonstrated a down-regulation of the *TEK* gene encoding Tie2 in oscillatory shear. Tie2 plays a central role in the regulation of endothelial barrier function. It has been shown that endothelial cells exposed to high shear stress have greater Tie2 activation and lower macromolecular permeability compared to areas with impaired blood flow [64]. In this case, arterial endothelial deletion of Tie2 is associated with progression of atherosclerosis, which corresponds to an increase in the number of inflammatory cells [65]. The current study demonstrated decreased TEK gene expression under oscillatory shear, which may contribute to atherogenesis.

Thus, hemodynamic abnormalities can lead to endothelial cell dysfunction and are associated with the development of vascular wall inflammation. Lipid mediators also play important roles in the development of vascular wall inflammation. The data obtained in the present study showed an increase in *ALOX5AP* expression during oscillatory flow, which may be another mechanism of hemodynamic and immunometabolic cross-linking. *ALOX5AP* (arachidonate 5-lipoxygenase-activating protein) is a gene that encodes a protein called 5-lipoxygenase activating protein (FLAP), which binds arachidonic acid and anchors 5-LOX to the membrane and plays an important role in the transfer of arachidonic acid to 5-LOX, thereby participating in the synthesis of proinflammatory leukotrienes that contribute to atherosclerosis [66,67,68,69]. The production of leukotrienes by activated leukocytes is an important part of the pathogenesis of atherosclerosis, since these mediators promote leukocyte adhesion to endothelial cells, smooth muscle cell proliferation, and foam cell formation. Additionally, leukotrienes induce the expression of proinflammatory cytokines and chemokines that contribute to the progression of atherosclerotic lesions. ALOX5AP expression has been shown to be elevated in human atherosclerotic plaques, promoting the accumulation of macrophages and other immune cells in arterial walls. In animal models, ALOX5AP deficiency reduces the size and complexity of atherosclerotic plaques, suggesting a critical role in the development of atherosclerosis. Importantly, FLAP is a nuclear membrane protein that is required for the synthesis of both leukotrienes and lipoxin A4 (LXA4)/resolvin D1 (RvD1), which are involved in the resolution of inflammation [70]. An imbalance between the production of pro-inflammatory leukotrienes and specialized pro-resolving mediators is an essential step in atherogenesis.

Thus, the current study strengthens the understanding that oscillatory flow has a significant effect on endothelial cells. Oscillatory shear affects the expression of genes related to the regulation of hemodynamics and immune and metabolic processes, which has implications for atherogenesis.

Smoking is an important risk factor for atherosclerosis. In the current study, cigarette smoke extract was associated with activation of inflammatory pathways including TNF signaling pathway, toll-like receptor signaling pathway, and NF-kappa B signaling pathway. The TNF pathway plays an important role in atherogenesis [71,72]. TNF has been shown to promote foam cell formation [73] and induce the expression of matrix metalloproteinases (MMPs) [74,75], enzymes that degrade the extracellular matrix and weaken the fibrous membrane of atherosclerotic plaques. This can lead to plaque rupture and the development of acute cardiovascular events. TNF is also implicated in the destabilization of vulnerable plaques [76], which are characterized by a thin fibrous sheath and a large necrotic core and are prone to rupture.

The NOD-like receptor signaling pathway is a key component of the innate immune system, and its activation has been implicated in the pathogenesis of atherosclerosis. NLRs are a family of cytoplasmic pattern recognition receptors that sense danger signals and initiate an inflammatory response. Upon activation, NLRs oligomerize and recruit adaptor proteins, leading to the activation of downstream signaling pathways, such as the NF-κB pathway. Activation of the NLR signaling pathway has been shown to contribute to the development of atherosclerosis by promoting lipid accumulation, endothelial dysfunction, and inflammation.

Prostaglandin-endoperoxide synthase 2 (PTGS2), also known as cyclooxygenase (COX)-2, plays an important role in the regulation of inflammation in the vascular wall [77]. COX-2 is expressed in endothelial cells, being expressed at low levels in quiescent cells, but its synthesis is rapidly induced by free radicals, cytokines, and growth factors [78]. COX-2 is responsible for the production of prostanoids involved in chronic inflammation and atherosclerosis. On the other hand, COX-2 may protect the cardiovascular system from atherosclerosis [79]. COX-2 may provide an additional pathway for the local production of protective prostacyclin [80]. In addition, global deletion of the COX-2 gene in mice contributes to the prothrombotic phenotype and leads to increased atherosclerosis [79,81,82,83]. This is due to the fact that the role of COX-2 in atherosclerosis is most likely related to the cell type and stage of atherosclerosis. The data obtained in the present study showed an increase in *PTGS2* gene expression after exposure to cigarette smoke extract.

Thus, smoking has been implicated in several inflammation-related signaling pathways, furthering our understanding of the role of this risk factor in atherogenesis. In addition, an increase in the expression of activating transcription factor 3 (*ATF3*) has been observed in endothelial cells exposed to cigarette smoke extract. ATF3 is a member of the family of transcription factors responsive to ATF/cAMP binding elements (CREB) [84,85]. ATF3 is maintained at low levels in quiescent cells, but its expression is induced by a variety of stresses. Treatment of human umbilical vein endothelial cells with TNF and oxLDL has been shown to induce ATF3. ATF3 has been implicated in the pathogenesis of atherosclerosis and may exert both inhibitory and stimulatory effects on the development of endothelial dysfunction [85]. Selective expression of ATF3 transcript variants under different conditions is thought to be responsible for a variety of ATF3 effects in the endothelium [85]. Atf3 has been identified as an important transcriptional regulator of regenerative responses in the arterial endothelium [86]. ATF3 expression was increased in atherosclerotic lesions where endothelial cells undergo programmed cell death [87,88]. ATF3 was shown to protect endothelial cells from TNF-induced apoptosis by suppressing p53 gene transcription, as ATF3 is a regulator of p53 protein stability and function [89,90]. At the same time, the cellular protective effect of ATF3 was significantly reduced in p53-deficient cells [90]. In addition, ATF3 has been shown to potentially mediate diabetic angiopathy [87]. Thus, ATF3 is involved in various vascular functions, including atherogenesis, through its role in the regulation of various processes related to hyperglycemia, dyslipidemia, and the immune response [85]. In this context, ATF3 is considered a novel prognostic biomarker and therapeutic target in atherosclerosis [91].

The results suggest that cigarette smoke extract affects immune mechanisms in endothelial cells, which may have implications in atherogenesis.

Another major risk factor for atherosclerosis, oxLDL, is involved in several mechanisms related to endothelial cell activation, dysfunction, and damage. These processes contribute to endothelial inflammation, endothelial barrier disruption, leukocyte adhesion, and endothelial cell senescence [92]. Accumulation of LDL beneath the endothelium is associated with several transport mechanisms. The contribution of passive filtration has been suggested to explain the increased lipid infiltration of the intima in areas of arteries with turbulent blood flow, where endothelial cells are not polarized and have loose intercellular contacts. The role of porous channels between adjacent cells of the continuous endothelial monolayer that allow transport of macromolecules has also been suggested. In addition, there is increasing evidence for the role of transcytosis, which may be receptor-mediated or receptor-independent [93,94].

The data obtained in the current study suggest that oxLDL may affect immune and metabolic processes in endothelial cells, including the NOD-like receptor pathway, the NF-κB pathway, and chemokine pathways.

The recruitment of immune cells to atherosclerotic lesions is another important step in the development and progression of atherosclerosis. A growing body of evidence supports the understanding of the role of chemokines in these processes. Endothelial cells release several chemokines to attract immune cells. In the present study, oxLDL increased the expression of *CX3CL1*, *CXCL10*, and *CXCL11*. Chemokine (C-X3-C motif) ligand 1 (CX3CL1) has chemotactic functions and mediates monocyte migration and adhesion. CXCL10, a member of the CXC family of chemokines, has diverse biological functions in different cell types, thus contributing to the initiation and progression of atherosclerosis [95]. CXCL11 shares a receptor with CXCL10, allowing them to perform similar functions. CXCL8, whose expression was up-regulated by smoking in the current study, may cause tight adhesion of rolling monocytes to the endothelial cell monolayer, contributing to the development of atherosclerosis [96]. On the other hand, the current study showed decreased *CCL2* gene expression when endothelial cells were exposed to cigarette smoke extract. CCL2, also known as monocyte chemoattractant protein-1 (MCP-1), is a potent inflammatory chemokine that regulates the migration and infiltration of a variety of immune cells, including monocytes and macrophages [97]. Previously, it was shown that exposure of HUVECs to nicotine and cigarette smoke condensate causes a significant decrease in MCP-1 expression [98]. At the same time, the expression of *CX3CL1* and *CCL2* was up-regulated in oscillatory shear, as shown in an additional analysis, whereas the expression of CXCL12 was higher in pulsatile flow. These findings underscore the multiple effects of impaired hemodynamics on endothelial function and atherogenesis. On the other hand, C-X-C chemokine receptor type 4 (*CXCR4*) expression was found to be increased during oscillatory shear. These results are interesting given the known evidence that the chemokine (C-X-C motif) ligand 12 (CXCL12)/CXCR4 axis supports endothelial barrier function through WNT/β-catenin, which regulates VE-cadherin signaling [99]. Increased activation of Wnt/β-catenin is known to contribute to endothelial dysfunction [100]. The release of inflammatory cytokines promotes activation of the Wnt signaling pathway, which may increase the progression of atherosclerosis and promote angiogenesis [101]. Thus, the Wnt pathway is associated with inflammation [102,103] These changes in chemokine expression may affect the maintenance of normal immune balance in the vascular wall.

This study has shown that the risk factors studied can have different effects on endothelial cells. It was also found that the risk factors studied can have different effects on the same genes, which further enhances the understanding of the complexity of the processes occurring in the vascular wall.

Thus, atherosclerosis is a complex chain of events developing in the vascular wall involving many cells, among which endothelial cells are key participants in vascular biology. In addition to the risk factors described above, insulin resistance and diabetes mellitus play an important role in atherogenesis [104]. It is important to note that NO production can be stimulated by insulin, whereas insulin resistance leads to impaired NO production [105]. Insulin resistance also contributes to elevated levels of proinflammatory markers and ROS, leading to increased intracellular levels of adhesion molecule-1 (ICAM-1) and VCAM-1 in endothelial cells [105,106].

It is important to note that the present study has some limitations, mainly related to the fact that the study did not include clinical or experimental confirmation of the findings but was based only on bioinformatic data analysis. In addition, the data were obtained with short-term exposure of endothelial cells to risk factors. Therefore, these data cannot be used to assess the long-term effects of such exposure. In addition, the data do not take into account the intercellular interactions that occur in the real vascular wall and play an important role in atherogenesis. In addition, the effect of single factors was assessed, and it is not possible to predict the complex effect of multiple factors. On the other hand, these data may be useful for planning future studies that may address these and other questions.

Because the data were obtained with short-term exposure of endothelial cells to risk factors, the findings may be useful in interpreting early changes in the vascular wall. In addition, the findings suggest an important role of endothelial cells in immune processes in atherosclerosis and underscore the key role of the endothelium in vascular homeostasis.

## 5. Conclusions

The results of this study provide important insights into the mechanisms underlying the development of atherosclerosis. Specifically, they suggest that atherogenic factors such as smoking, impaired flow, and oxLDL may lead to impaired innate immune response, metabolism, and apoptosis in endothelial cells. This, in turn, may contribute to the progression of atherosclerosis.

The findings may advance our understanding of the molecular mechanisms underlying these risk factors, including smoking and dyslipidemia, to identify potential therapeutic targets and develop effective preventive strategies. Thus, a better understanding of the pathophysiology of atherosclerosis may allow the development of new approaches to reduce the prevalence and severity of this disease.

Furthermore, this study highlights the important role of the endothelium in vascular homeostasis and the potential for endothelial dysfunction to contribute to the development of atherosclerosis. Future studies should investigate the complex intercellular interactions that occur in the real vascular wall, as well as the long-term effects of exposure to atherogenic risk factors. Such studies may contribute to the development of more targeted and effective prevention and treatment strategies for atherosclerosis.

## Figures and Tables

**Table 1:** Functional roles and general information of the down-regulated hub genes.

Gene Symbol	Other Names	Full Name	Protein	Biological Processes
*CRP*	*PTX1*	C-reactive protein	C-reactive protein	Inflammatory response; innate immune response; negative regulation of macrophage-derived foam cell differentiation.
*TEK*	*TIE2*; *VMCM*; *GLC3E*; *TIE-2*; *VMCM1*; *CD202B*	TEK receptor tyrosine kinase	Angiopoietin-1 receptor	Angiogenesis; endothelial cell proliferation; cell–cell signaling; negative regulation of endothelial cell apoptotic process; negative regulation of apoptotic process; positive regulation of endothelial cell migration; positive regulation of MAPK cascade; positive regulation of actin cytoskeleton reorganization; regulation of vascular permeability.
*NTRK1*	*MTC*; *TRK*; *TRK1*; *TRKA*; *Trk-A*; *p140-TrkA*	Neurotrophic receptor tyrosine kinase 1	High-affinity nerve growth factor receptor	Negative regulation of apoptotic process; positive regulation of programmed cell death; mechanoreceptor differentiation; positive regulation of angiogenesis; positive regulation of ERK1 and ERK2 cascade; positive regulation of NF-kappaB transcription factor activity; transmembrane receptor protein tyrosine kinase signaling pathway.
*CD34*	-	CD34 molecule	Hematopoietic progenitor cell antigen	Cell motility; cell–cell adhesion; cell–matrix adhesion; endothelial cell proliferation; hemopoiesis; leukocyte migration; negative regulation of blood coagulation; positive regulation of angiogenesis.
*FGF18*	*ZFGF5*; *FGF-18*	Fibroblast growth factor 18	Fibroblast growth factor 18	Angiogenesis; cell differentiation; cell–cell signaling; ERK1 and ERK2 cascade; positive regulation of blood vessel endothelial cell migration; positive regulation of endothelial cell chemotaxis to fibroblast growth factor.
*NOS3*	*eNOS*; *ECNOS*	Nitric oxide synthase 3	Nitric oxide synthase, endothelial	Nitric oxide biosynthetic process; angiogenesis; endothelial cell migration; negative regulation of blood pressure; regulation of platelet activation; negative regulation of smooth muscle cell proliferation.
*KLF4*	*EZF*; *GKLF*	KLF transcription factor 4	Krueppel-like factor 4	Cellular response to laminar fluid shear stress; canonical Wnt signaling pathway; negative regulation of angiogenesis; negative regulation of inflammatory response; negative regulation of interleukin-8 production; negative regulation of leukocyte adhesion to arterial endothelial cell; negative regulation of NF-kappaB transcription factor activity; negative regulation of response to cytokine stimulus.
*THBD*	*TM*; *THRM*; *AHUS6*; *BDCA3*; *CD141*; *BDCA-3*; *THPH12*	Thrombomodulin	Thrombomodulin	Blood coagulation; negative regulation of blood coagulation; negative regulation of platelet activation.
*ITPR3*	*IP3R*; *CMT1J*; *IP3R3*	Inositol 1,4,5-trisphosphate receptor type 3	Inositol 1,4,5-trisphosphate receptor type 3	G protein-coupled receptor signaling pathway; calcium transport.
*ELN*	*WS*; *WBS*; *SVAS*; *ADCL1*	Elastin	Elastin	Extracellular matrix assembly; regulation of smooth muscle cell proliferation.

**Table 2:** Functional roles and general information of the up-regulated hub genes.

Gene Symbol	Other Names	Full Name	Protein	Biological Processes
*ALOX5AP*	*FLAP*	Arachidonate 5-lipoxygenase-activating protein	Arachidonate 5-lipoxygenase-activating protein	Leukotriene biosynthesis; synthesis of lipoxins (LX); synthesis of 5-eicosatetraenoic acids.
*ANGPT2*	*ANG2*; *AGPT2*; *LMPHM10*	Angiopoietin 2	Angiopoietin-2	Angiogenesis; response to glucose; response to mechanical stimulus; response to hypoxia.
*KIT*	*PBT*; *SCFR*; *C-Kit*; *CD117*; *MASTC*	KIT proto-oncogene, receptor tyrosine kinase	Mast/stem cell growth factor receptor kit	Regulation of cell survival and proliferation; mast cell development, migration, and function; actin cytoskeleton reorganization; hematopoiesis, stem cell maintenance; cytokine-mediated signaling pathway.
*LCP2*	*IMD81*; *SLP76*; *SLP-76*	Lymphocyte cytosolic protein 2	Lymphocyte cytosolic protein 2	Immune response; intracellular signal transduction; mast cell activation; positive regulation of protein kinase activity; transmembrane receptor protein tyrosine kinase signaling pathway.
*C3AR1*	*AZ3B*; *C3AR*; *HNFAG09*	Complement C3a receptor 1	C3a anaphylatoxin chemotactic receptor	Chemotaxis; positive regulation of macrophage chemotaxis; complement receptor-mediated signaling pathway; inflammatory response; positive regulation of angiogenesis; positive regulation of vascular endothelial growth factor production.
*CD69*	*AIM*; *EA1*; *MLR-3*; *CLEC2C*; *GP32/28*; *BL-AC/P26*	CD69 molecule	Early-activation antigen CD69	Cellular response to xenobiotic stimulus.
*CXCR4*	*FB22*; *HM89*; *LAP3*; *LCR1*; *NPYR*; *WHIM*; *CD184*; *LAP-3*; *LESTR*; *NPY3R*; *NPYRL*; *WHIMS*; *HSY3RR*; *NPYY3R*; *WHIMS1*; *D2S201E*	C-X-C motif chemokine receptor 4	C-X-C chemokine receptor type 4	Apoptotic process; cell chemotaxis; CXCL12-activated CXCR4 signaling pathway; endothelial cell differentiation; immune response; inflammatory response; positive regulation of cell migration.

**Table 3:** Functional roles and general information of the up-regulated hub genes.

Gene Symbol	Other Names	Full Name	Protein	Biological Processes
*EGR1*	*TIS8*; *AT225*; *G0S30*; *NGFI-A*; *ZNF225*; *KROX-24*; *ZIF-268*	Early-growth response 1	Early-growth response protein 1	Cellular response to interleukin-8; interleukin-1-mediated signaling pathway; negative regulation of canonical Wnt signaling pathway; positive regulation of chemokine production; regulation of apoptotic process; response to glucose; response to insulin; response to hypoxia.
*ATF3*	-	Activating transcription factor 3	Cyclic AMP-dependent transcription factor ATF-3	Gluconeogenesis; positive regulation of cell population proliferation; positive regulation of transcription by RNA polymerase II.
*CXCL8*	*IL8*; *NAF*; *GCP1*; *LECT*; *LUCT*; *NAP1*; *GCP-1*; *LYNAP*; *MDNCF*; *MONAP*; *NAP-1*; *SCYB8*	C-X-C motif chemokine ligand 8	Interleukin-8	Chemotaxis; neutrophil chemotaxis; angiogenesis; cellular response to interleukin-1; cellular response to lipopolysaccharide; inflammatory response; regulation of cell adhesion.
*SOCS3*	*CIS3*; *SSI3*; *ATOD4*; *Cish3*; *SSI-3*; *SOCS-3*	Suppressor of cytokine signaling 3	Suppressor of cytokine signaling 3	Cell differentiation; negative regulation of apoptotic process; negative regulation of inflammatory response; negative regulation of insulin receptor signaling pathway.
*FOS*	*p55*; *AP-1*; *C-FOS*	Fos proto-oncogene, AP-1 transcription factor subunit	Protein c-Fos	Cellular response to hypoxia; response to insulin; cellular response to reactive oxygen species; cellular response to tumor necrosis factor; inflammatory response; response to toxic substance; response to xenobiotic stimulus.
*PTGS2*	*COX2*; *COX-2*; *PHS-2*; *PGG/HS*; *PGHS-2*; *hCox-2*; *GRIPGHS*	Prostaglandin-endoperoxide synthase 2	Prostaglandin G/H synthase 2	Angiogenesis; cellular response to fluid shear stress; cellular response to hypoxia; inflammatory response; cyclooxygenase pathway; prostaglandin biosynthetic process; positive regulation of vasoconstriction; fatty acid metabolism.

**Table 4:** Functional roles and general information of the down-regulated hub genes.

Gene Symbol	Other Names	Full Name	Protein	Biological Processes
*MX1*	*MX*; *MxA*; *IFI78*; *IFI-78K*; *lncMX1-215*	MX dynamin like GTPase 1	Interferon-induced GTP-binding protein Mx1	Antiviral innate immune response; apoptotic process; innate immune response; response to Type I interferon.
*CMPK2*	*NDK*; *TYKi*; *TMPK2*; *UMP-CMPK2*	Cytidine/uridine monophosphate kinase 2	UMP-CMP kinase 2, mitochondrial	Cellular response to lipopolysaccharide; pyrimidine biosynthesis.
*OAS1*	*OIAS*; *IFI-4*; *OIASI*; *IMD100*; *E18/E16*	2′-5′-oligoadenylate synthetase 1	2′-5′-oligoadenylate synthase 1	Cellular response to interferon-alpha; cellular response to interferon-beta; glucose homeostasis; antiviral defense.
*OAS2*	*-*	2′-5′-oligoadenylate synthetase 2	2′-5′-oligoadenylate synthase 2	Antiviral defense; immunity; innate immunity.
*IFI44L*	*GS3686*; *TLDC5B*; *C1orf29*	Interferon-induced protein 44 like	Interferon-induced protein 44-like	Antiviral defense.
*IFIH1*	*-*	Interferon-induced with helicase C domain 1	Interferon-induced helicase C domain-containing protein 1	Antiviral defense; immunity; innate immunity.
*PARP9*	*BAL*; *BAL1*; *ARTD9*; *MGC:7868*	Poly(ADP-ribose) polymerase family member 9	Protein mono-ADP-ribosyltransferase PARP9	Antiviral defense; immunity; innate immunity; DNA damage; DNA repair.
*RSAD2*	*SAND*; *cig5*; *vig1*; *cig33*	Radical S-adenosyl methionine domain containing 2	S-adenosylmethionine-dependent nucleotide dehydratase RSAD2	Antiviral defense; immunity; innate immunity.
*XAF1*	*BIRC4BP*; *XIAPAF1*; *HSXIAPAF1*	XIAP-associated factor 1	XIAP-associated factor 1	Apoptotic process; response to interferon-beta.
*IFIT1*	*C56*; *P56*; *G10P1*; *IFI56*; *ISG56*; *IFI-56*; *IFIT-1*; *IFNAI1*; *RNM561*; *IFI-56K*	Interferon-induced protein with tetratricopeptide repeats 1	Interferon-induced protein with tetratricopeptide repeats 1	Antiviral defense; immunity; innate immunity.

**Table 5:** Functional roles and general information of the up-regulated hub genes.

Gene Symbol	Other Names	Full Name	Protein	Biological Processes
*MX1*	*MX*; *MxA*; *IFI78*; *IFI-78K*; *lncMX1-215*	MX dynamin like GTPase 1	Interferon-induced GTP-binding protein Mx1	Antiviral innate immune response; apoptotic process; innate immune response; response to Type I interferon.
*IFIH1*	*-*	Interferon-induced with helicase C domain 1	Interferon-induced helicase C domain-containing protein 1	Antiviral defense; immunity; innate immunity.
*IFIT1*	*C56*; *P56*; *G10P1*; *IFI56*; *ISG56*; *IFI-56*; *IFIT-1*; *IFNAI1*; *RNM561*; *IFI-56K*	Interferon-induced protein with tetratricopeptide repeats 1	Interferon-induced protein with tetratricopeptide repeats 1	Antiviral defense; immunity; innate immunity.
*OAS2*	*-*	2′-5′-oligoadenylate synthetase 2	2′-5′-oligoadenylate synthase 2	Antiviral defense; immunity; innate immunity.
*OAS3*	*p100*; *p100OAS*	2′-5′-oligoadenylate synthetase 3	2′-5′-oligoadenylate synthase 3	Antiviral defense; immunity; innate immunity.
*DDX58*	*RIGI*; *RIG1*; *RIG-I*; *RLR-1*; *SGMRT2*	RNA sensor RIG-I	Antiviral innate immune response receptor RIG-I	Antiviral defense; immunity; innate immunity.
*ISG15*	*G1P2*; *IP17*; *UCRP*; *IFI15*; *IMD38*; *hUCRP*	ISG15 ubiquitin-like modifier	Ubiquitin-like protein ISG15	Antiviral defense; immunity; innate immunity.
*OASL*	*OASL1*; *OASLd*; *TRIP14*; *TRIP-14*; *p59OASL*; *p59 OASL*; *p59-OASL*	2′-5′-oligoadenylate synthetase-like	2′-5′-oligoadenylate synthase-like protein	Antiviral defense; immunity; innate immunity.
*IFIT2*	*P54*; *G10P2*; *IFI54*; *ISG54*; *cig42*; *IFI-54*; *IFIT-2*; *ISG-54*; *GARG-39*; *IFI-54K*; *ISG-54K*; *ISG-54 K*	Interferon-induced protein with tetratricopeptide repeats 2	Interferon-induced protein with tetratricopeptide repeats 2	Antiviral defense; immunity; innate immunity.

**Table 6:** Functional roles and general information of the down-regulated hub genes.

Gene Symbol	Other Names	Full Name	Protein	Biological Processes
*IDI1*	*IPP1*; *IPPI1*	Isopentenyl-diphosphate delta isomerase 1	Isopentenyl-diphosphate Delta-isomerase 1	Cholesterol biosynthetic process isoprenoid biosynthetic process; lipid metabolism.
*INSIG1*	*CL6*	Insulin-induced gene 1	Insulin-induced gene 1 protein	Cellular response to insulin stimulus; cellular response to sterol; cholesterol biosynthetic process; SREBP signaling pathway; triglyceride metabolic process.
*LDLR*	*FH*; *FHC*; *FHCL1*; *LDLCQ2*	Low-density lipoprotein receptor	Low-density lipoprotein receptor	Artery morphogenesis; cellular response to fatty acid; cellular response to low-density lipoprotein particle stimulus; cholesterol homeostasis; endocytosis; lipid metabolic process; low-density lipoprotein particle clearance; phagocytosis; phospholipid transport; positive regulation of inflammatory response; receptor-mediated endocytosis.
*SREBF2*	*SREBP2*; *bHLHd2*; *SREBP-2*	Sterol regulatory element-binding transcription factor 2	Sterol regulatory element-binding protein 2	Cellular response to laminar fluid shear stress; cholesterol homeostasis; lipid metabolic process; sterol metabolism.
*DHCR24*	*DCE*; *SELADIN1*; *Nbla03646*; *seladin-1*	24-dehydrocholesterol reductase	Delta(24)-sterol reductase	Cholesterol biosynthetic process; membrane organization; negative regulation of apoptotic process; response to oxidative stress; steroid biosynthesis.
*HMGCS1*	*HMGCS*	3-hydroxy-3-methylglutaryl-CoA synthase 1	Hydroxymethylglutaryl-CoA synthase, cytoplasmic	Cholesterol biosynthetic process; lipid metabolic process.
*ACAT2*	*-*	Acetyl-CoA acetyltransferase 2	Acetyl-CoA acetyltransferase, cytosolic	Acetyl-CoA C-acetyltransferase activity; fatty acid beta-oxidation; lipid metabolic process.
*SQLE*	*-*	Squalene epoxidase	Squalene monooxygenase	Cholesterol metabolic process; sterol biosynthetic process; regulation of cell population proliferation.

## Data Availability

The data presented in this study are available on request from the corresponding author.

## Supplementary Materials

The following supporting information can be downloaded at: https://www.mdpi.com/article/10.3390/biomedicines11041216/s1, Figure S1: Network of up-regulated DEGs protein-protein interactions from data set GSE103672; Figure S2: Network of down-regulated DEGs protein-protein interactions from data set GSE103672; Figure S3: Network of up-regulated DEGs protein-protein interactions from data set GSE141136 Figure S4: Network of down-regulated DEGs protein-protein interactions from data set GSE141136; Figure S5: Network of up-regulated DEGs protein-protein interactions from data set GSE29881; Figure S6: Network of down-regulated DEGs protein-protein interactions from data set GSE29881.
