# Supplementary figures and images for: Effects of Atherogenic Factors on Endothelial Cells: Bioinformatics Analysis of Differentially Expressed Genes and Signaling Pathways
Source: Biomedicines. 2023 Apr 19;11(4):1216. doi: 10.3390/biomedicines11041216 (PMC10135807; doi:10.3390/biomedicines11041216)

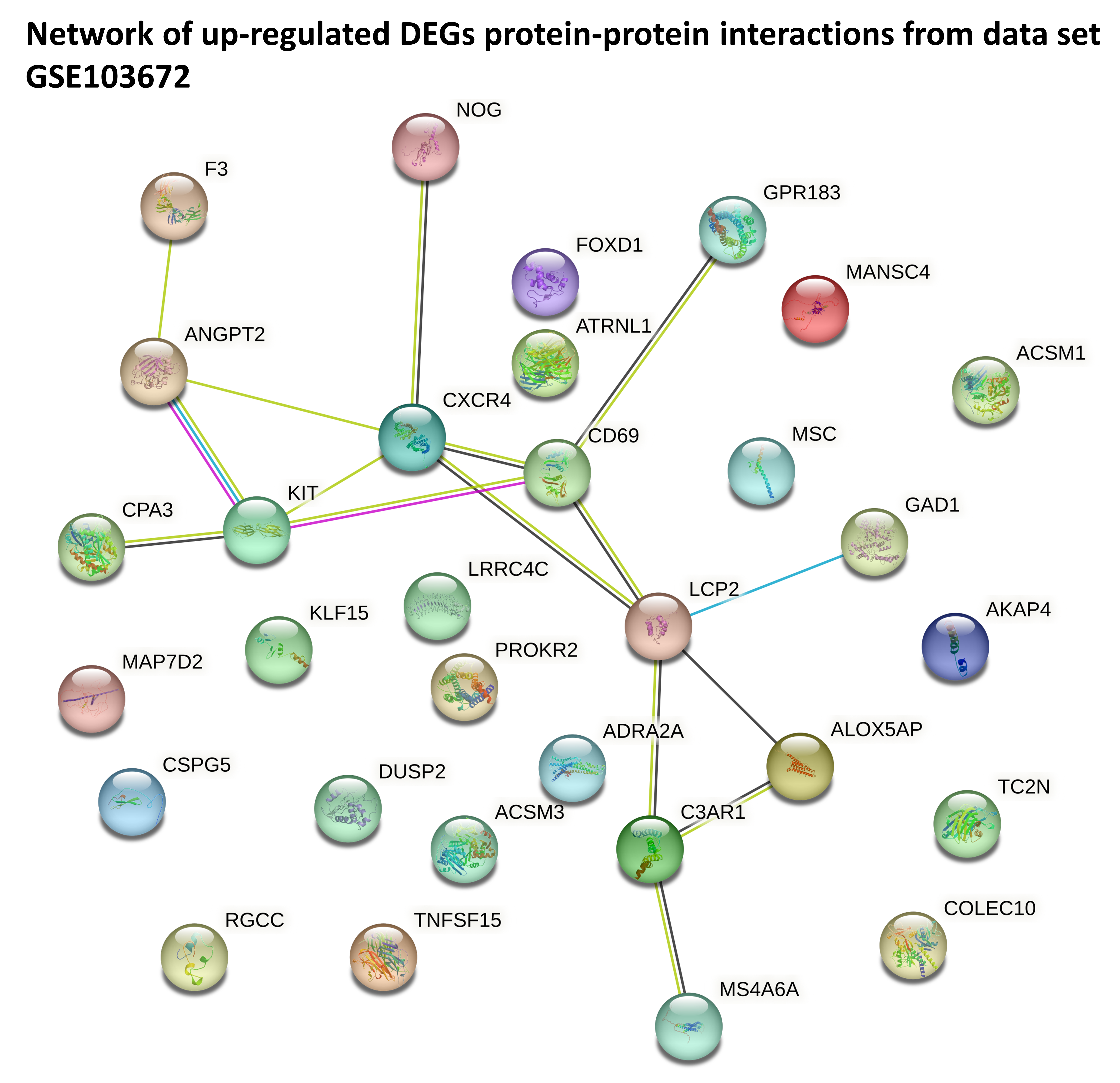

Supplement: Supplementary file 1 [file biomedicines-11-01216-s001.zip › Suppl figure S1.tif]

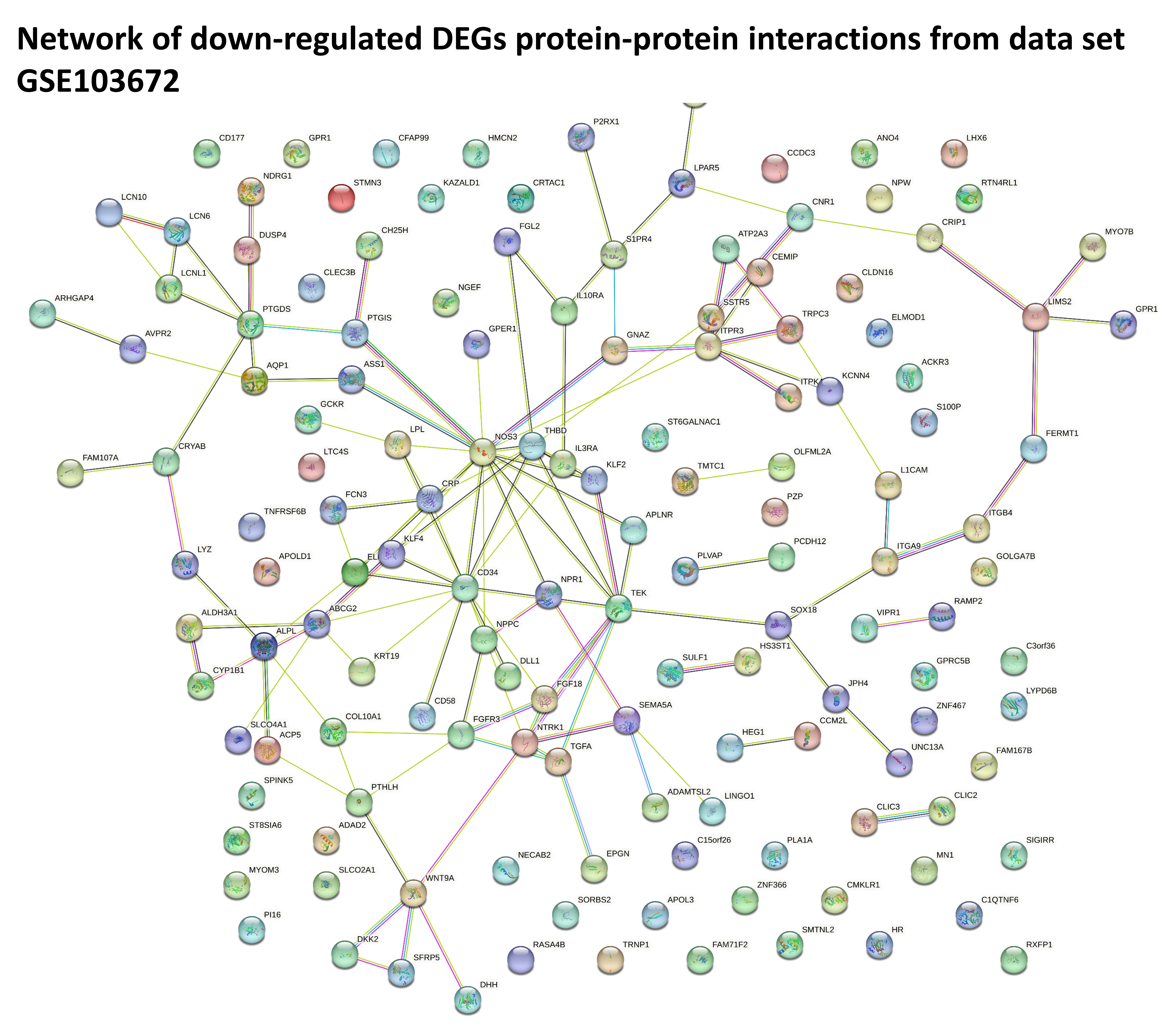

Supplement: Supplementary file 1 [file biomedicines-11-01216-s001.zip › Suppl figure S2.tif]

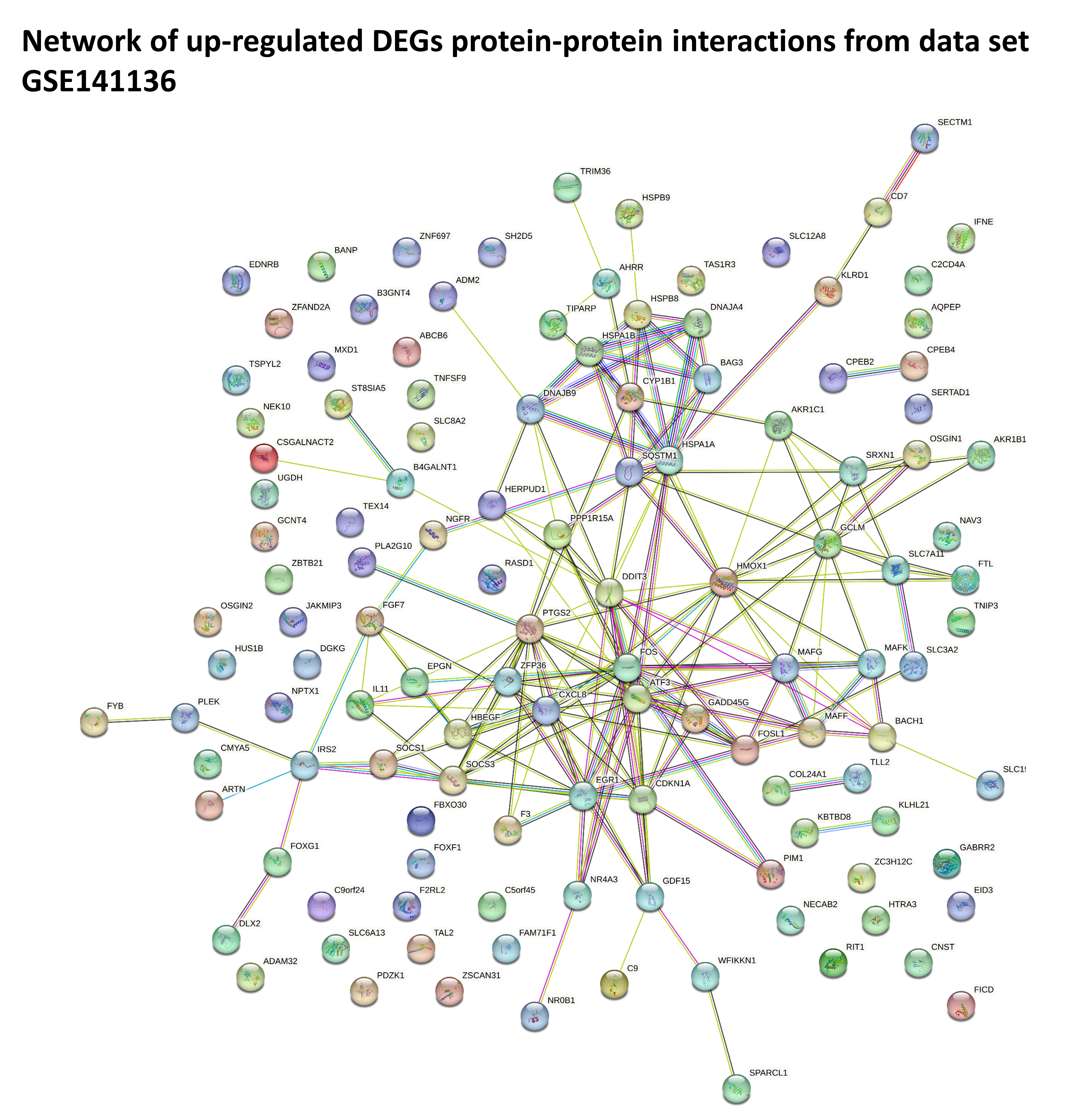

Supplement: Supplementary file 1 [file biomedicines-11-01216-s001.zip › Suppl figure S3.tif]

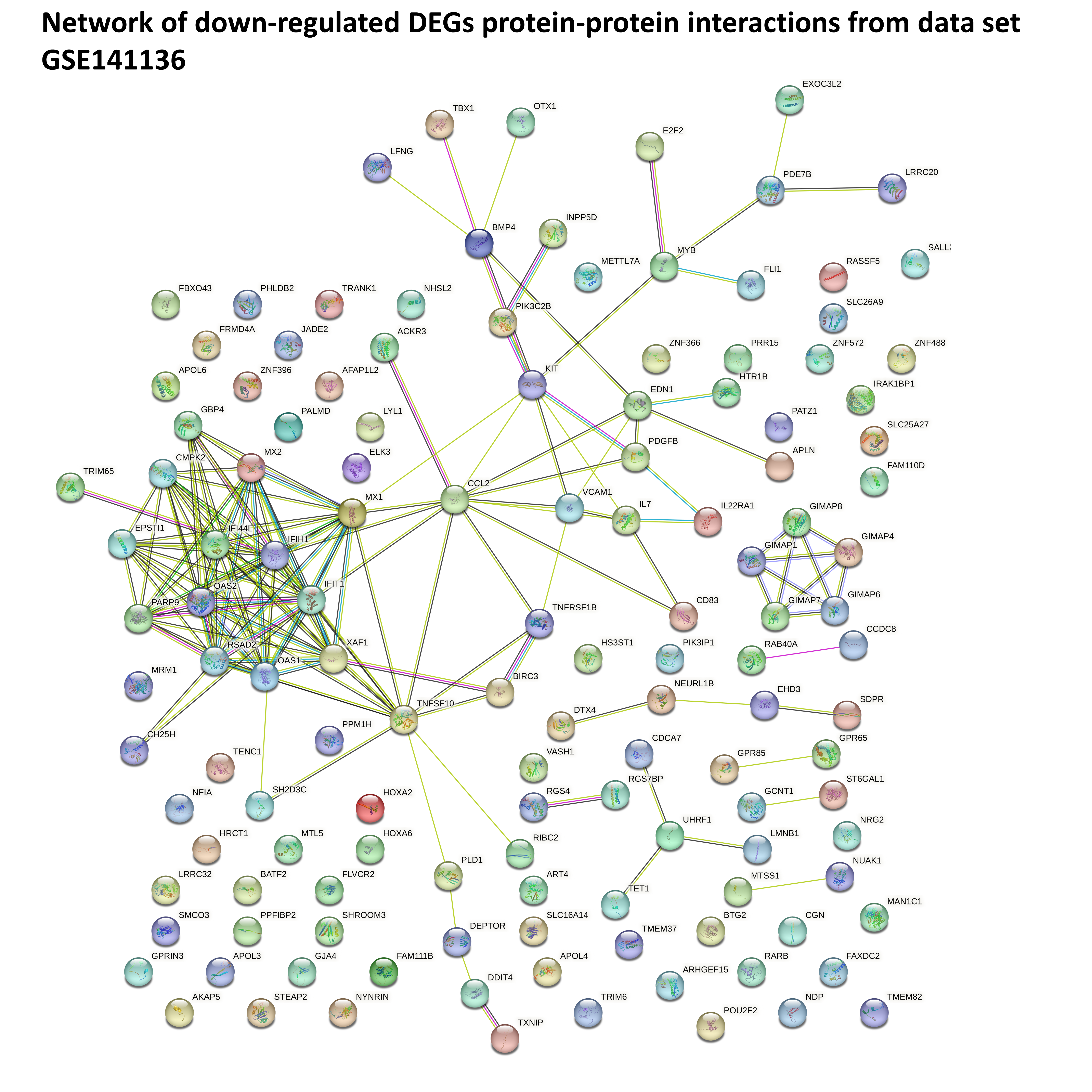

Supplement: Supplementary file 1 [file biomedicines-11-01216-s001.zip › Suppl figure S4.tif]

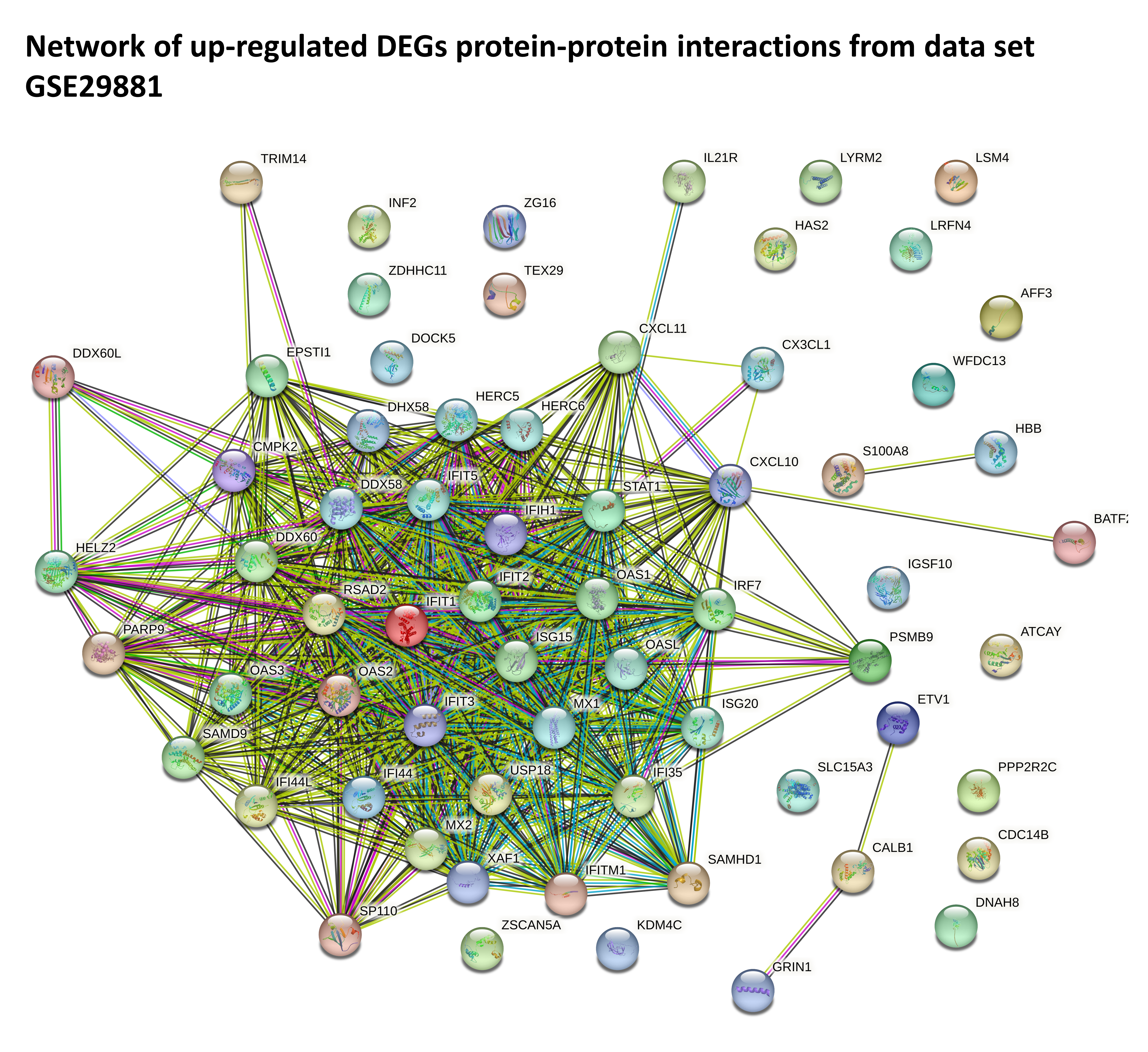

Supplement: Supplementary file 1 [file biomedicines-11-01216-s001.zip › Suppl figure S5.tif]

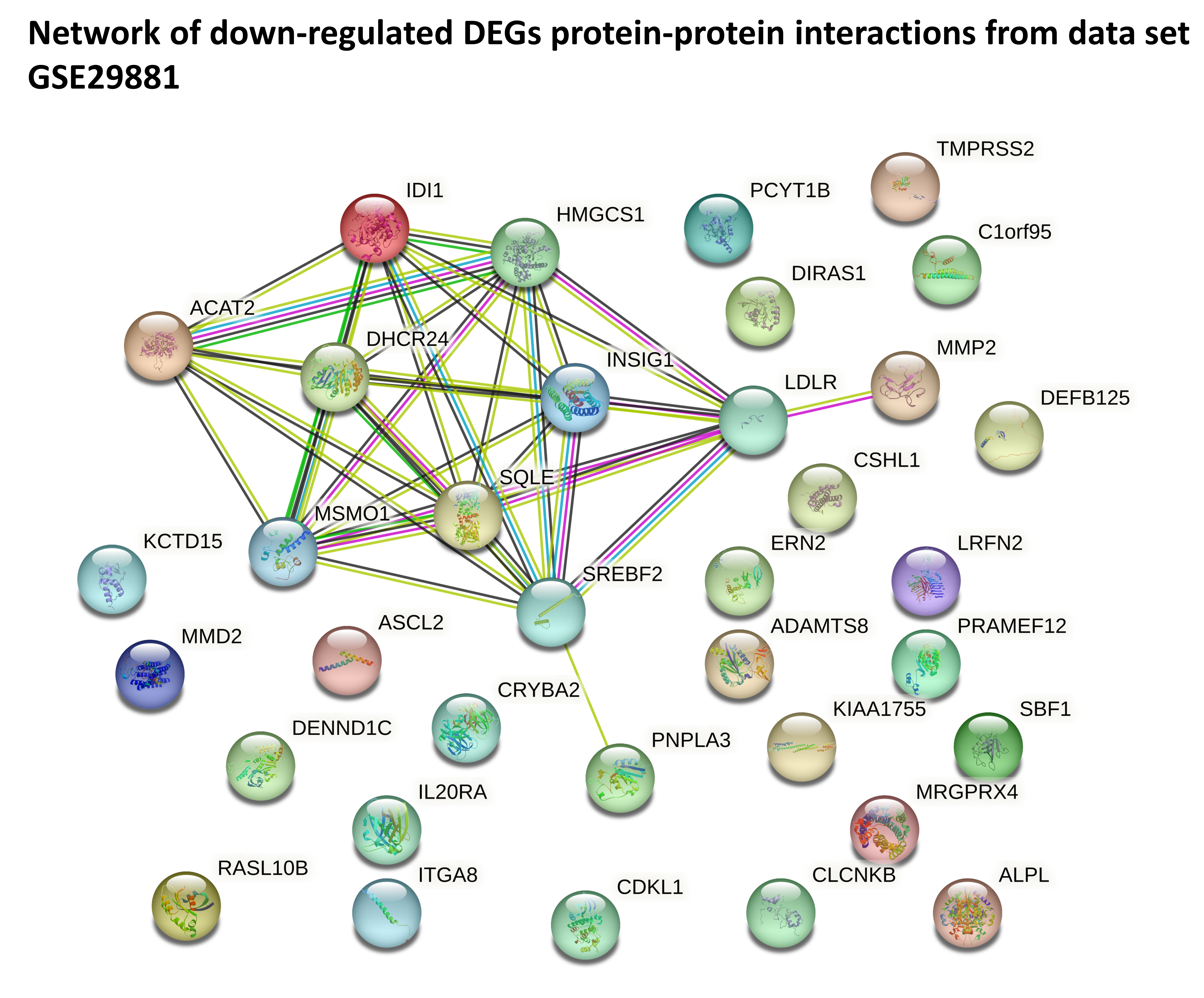

Supplement: Supplementary file 1 [file biomedicines-11-01216-s001.zip › Suppl figure S6.tif]
